# Carotid geometry is an independent predictor of wall thickness – a 3D cardiovascular magnetic resonance study in patients with high cardiovascular risk

**DOI:** 10.1186/s12968-020-00657-5

**Published:** 2020-09-10

**Authors:** Christoph Strecker, Axel Joachim Krafft, Lilli Kaufhold, Markus Hüllebrandt, Susanne Weber, Ute Ludwig, Martin Wolkewitz, Anja Hennemuth, Jürgen Hennig, Andreas Harloff

**Affiliations:** 1 Department of Neurology and Neurophysiology, Medical Center - University of Freiburg, Faculty of Medicine, University of Freiburg, Breisacherstrasse 64, 79106 Freiburg, Germany; 2 Department of Radiology - Medical Physics, Medical Center - University of Freiburg, Faculty of Medicine, University of Freiburg, Freiburg, Germany; 3 Fraunhofer MEVIS, Bremen, Germany; 4 Institute for Imaging Science and Computational Modelling in Cardiovascular Medicine, Charité-Universitätsmedizin Berlin, Berlin, Germany; 5 Institute of Medical Biometry and Statistics, Faculty of Medicine and Medical Center, University of Freiburg, Freiburg, Germany

**Keywords:** Carotid artery, Atherosclerosis, Wall shear stress, Carotid geometry, Magnetic resonance imaging

## Abstract

**Background:**

The posterior wall of the proximal internal carotid artery (ICA) is the predilection site for the development of stenosis. To optimally prevent stroke, identification of new risk factors for plaque progression is of high interest. Therefore, we studied the impact of carotid geometry and wall shear stress on cardiovascular magnetic resonance (CMR)-depicted wall thickness in the ICA of patients with high cardiovascular disease risk.

**Methods:**

One hundred twenty-one consecutive patients ≥50 years with hypertension, ≥1 additional cardiovascular risk factor and ICA plaque ≥1.5 mm thickness and < 50% stenosis were prospectively included. High-resolution 3D-multi-contrast (time of flight, T1, T2, proton density) and 4D flow CMR were performed for the assessment of morphological (bifurcation angle, ICA/common carotid artery (CCA) diameter ratio, tortuosity, and wall thickness) and hemodynamic parameters (absolute/systolic wall shear stress (WSS), oscillatory shear index (OSI)) in 242 carotid bifurcations.

**Results:**

We found lower absolute/systolic WSS, higher OSI and increased wall thickness in the posterior compared to the anterior wall of the ICA bulb (*p* < 0.001), whereas this correlation disappeared in ≥10% stenosis. Higher carotid tortuosity (regression coefficient = 0.764; *p* < 0.001) and lower ICA/CCA diameter ratio (regression coefficient = − 0.302; *p* < 0.001) were independent predictors of increased wall thickness even after adjustment for cardiovascular risk factors. This association was not found for bifurcation angle, WSS or OSI in multivariate regression analysis.

**Conclusions:**

High carotid tortuosity and low ICA diameter were independent predictors for wall thickness of the ICA bulb in this cross-sectional study, whereas this association was not present for WSS or OSI. Thus, consideration of geometric parameters of the carotid bifurcation could be helpful to identify patients at increased risk of carotid plaque generation. However, this association and the potential benefit of WSS measurement need to be further explored in a longitudinal study.

## Background

Internal carotid artery (ICA) stenoses are one of the major sources of ischemic stroke [1]. They are attributed to traditional cardiovascular risk factors acting on the arterial wall over decades. However, ICA stenoses often develop asymmetrically [2], suggesting that additional parameters such as carotid geometry and the specific distribution of wall shear stress (WSS) may be responsible for the development and progression of atherosclerosis [3]. This is further supported by the observation that atherosclerosis is particularly concentrated at the outlet of arteries such as the carotid bulb while straight segments such as the common carotid artery or abdominal aorta are far less affected [4–6]. This suggests that hemodynamic, geometric or genetic factors play an important role at arterial bifurcations or curvatures while cardiovascular risk factors are the dominant factor in straight vessels [7]. Previous studies have shown that individual geometry, low WSS and high oscillatory shear index (OSI) at the carotid bifurcation could play a role in the development of atherosclerotic lesions [7–9]. Studies in healthy subjects using computational fluid dynamics (CFD) and 4D flow cardiovascular magnetic resonance (CMR) demonstrated that these potentially atherogenic flow conditions predominantly occur at the posterior wall of the proximal ICA (bulb) [4, 8, 10]. Interestingly, this coincides with the area where atherosclerotic plaques and stenoses typically occur [7, 8, 10]. Furthermore, animal models revealed that WSS correlated with plaque composition, leading to unstable “high-risk” plaques in zones with low WSS [11, 12]. Low WSS and high OSI obviously initiate atherosclerosis through endothelial damage resulting in increased susceptibility to wall injury and inflammation. Accordingly, this facilitates lipids to migrate into the vessel wall. By contrast, there is emerging evidence that high WSS possibly promotes intra-plaque hemorrhage, thinning of the fibrous cap and ultimately leads to plaque rupture [3, 7, 13, 14].

Bifurcation geometry was proposed as an important factor for these hemodynamic conditions and considered as a potential surrogate parameter for atherosclerotic risk prediction. The analysis of computed tomography angiography (CTA) in 178 patients by Phan et al. [15] underlined that geometry and anatomy enhance the risk of carotid stenosis independent of traditional vascular risk factors. This was confirmed by Bijari et al. [16], who studied bifurcation geometry and wall thickness using CMR in > 1000 patients. However, three-dimensional (3D) blood flow, WSS and OSI were not investigated in either study. In turn, a study of 14 subjects, in which WSS was measured by CFD and 4D flow CMR, revealed that wall thickness at the bifurcation increased with decreasing WSS [17]. However, the number of subjects was small and individual carotid geometry was not considered. Thus, to date there is no larger study that has investigated patients with carotid plaques by measuring the impact of both carotid geometry and WSS on carotid wall thickness.

Therefore, to study this fluid-structure interaction, we applied a comprehensive 3D-multi-contrast CMR protocol in a larger cohort of high-risk patients with carotid atherosclerosis and fused information on individual carotid geometry, WSS and OSI with wall thickness data at the ICA bulb. To study patients with atherosclerosis of various degrees we included small plaques up to 50% ICA stenosis and applied a plane- and segment-wise analysis strategy which is well suited for carotid wall thickness measurement and which was used as one outcome variable in the present study. We intended to identify independent risk factors for carotid atherosclerosis beyond traditional cardiovascular risk factors that can be used to detect high-risk patients for carotid artery atherosclerosis and to optimize their therapy in the future.

## Methods

### Study population

From April 2018 to February 2019, all patients from our in- and outpatient clinic undergoing carotid ultrasound were consecutively and prospectively screened for eligibility. Inclusion criteria were: ≥50 years of age, arterial hypertension and at least one additional risk factor (smoking, diabetes mellitus, hyperlipidemia, peripheral arterial disease (PAD), coronary artery disease (CAD), history of ischemic stroke or transient ischemic attack (TIA)) and evidence of at least one 1.5 mm thick plaque of the ICA or the distal common carotid artery (CCA) in ultrasound.

Exclusion criteria were: contraindications to 3 T CMR such as ferromagnetic implants, claustrophobia, poor clinical condition (modified ranking scale (mRS) > 3), atrial fibrillation or other relevant cardiac arrhythmias interfering with the electrocardiographic (ECG)-trigger), ICA-stenosis > 50% or ICA-occlusion according to North American Symptomatic Carotid Endarterectomy Trial (NASCET) criteria [18], expectation of life < 2 years, pregnancy, distance to place of residence > 100 km and refusal of study participation.

**Fig. 1:**
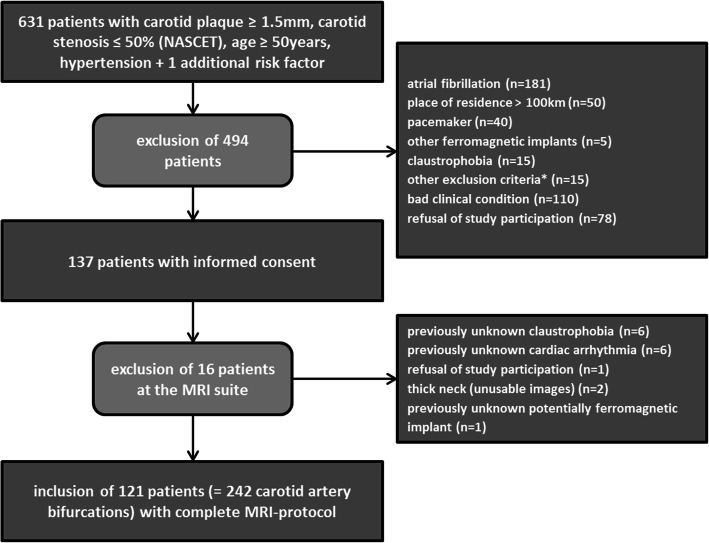
Recruitment algorithm of the study cohort; *to obese for CMR scanner (6), expectation of life < 2 years (7), unable to lie in a supine position due to back pain (2)

Six hundred and thirty-one patients fulfilled the inclusion criteria of which 494 were excluded (Fig. 1) leaving 121 patients for analysis. Patients’ demographics and cardiovascular disease risk factors were obtained from patients’ electronic charts and personal interviews before CMR study.

Written informed consent was obtained from all participants. The local ethics committee approved the study and all ultrasound and CMR procedures were in accordance with institutional guidelines.

### Ultrasound examination

All patients underwent 2D Duplex sonography of extracranial and intracranial arteries (IU22, Philips Healthcare, Bothwell, Washington, USA). A linear array probe (L 9–3 MHz, Philips Healthcare) was used for extracranial and a curved array probe (S 5–1 MHz, Philips Healthcare) for intracranial examination. Ultrasound was performed by two medical technical assistants with experience in neurovascular ultrasound > 20 years or by a physician with several years of ultrasound experience. Patients were examined in a supine position and maximum wall thickness and plaque thickness were measured manually by electronic calipers at the far wall of the left and right CCA and ICA. ICA stenosis was graded according to NASCET criteria based on 2D Duplex sonography [18].

### CMR-protocol

Multi-contrast CMR included 3D time of flight (TOF), T1-, T2-, and proton density (PD)-weighted imaging. For T1-, T2- and PD-weighted imaging a variable-flip-angle 3D turbo spin echo (TSE) sequence (Sampling Perfection with Application optimized Contrasts using different flip angle Evolution–SPACE) with fat saturation and dark-blood preparation was used. Imaging was executed on a 3 T CMR scanner (Prisma, Siemens Healthineers, Erlangen, Germany) with an 8-channel surface coil (NORAS MRI products GmbH, Hoechberg, Germany).

An isotropic spatial resolution of 0.6mm^3^ was used for all sequences except for 3D-TOF (0.5 × 0.5 × 0.6 mm). Other sequence parameters were: *3D-TOF:* repetition time (TR)/echo time (TE) = 21/3.3 ms, field of view (FOV) = 180 × 135 mm^2^, matrix 384 × 290, flip angle = 20°, pixel bandwidth (BW) = 250 Hz/Px, total acquisition time (TA) = 4:32 min. *T1-weighted SPACE:* TR/TE = 900/26 ms, BW = 405 Hz/Px, echo train length (ETL) = 36, TA = 7:32 min. *T2-weighted SPACE:* TR/TE = 2000/159 ms, BW = 405 Hz/Px, ETL = 61, TA = 7:22 min. *PD-weighted SPACE:* TR/TE = 1900/26 ms, BW = 405 Hz/Px, ETL = 61, TA = 8:04 min.

*4D flow* data (spatial/temporal resolution = 0.8 mm^3^/52.8 ms) were acquired with prospective ECG-triggering using a k-t-accelerated time-resolved 3D phase contrast sequence [19, 20]. Other sequence parameters were: TR/TE = 52.8/3.9 ms, flip angle = 12°, peak GRAPPA acceleration factor = 5, FOV = 140x140mm^2^, slice thickness = 0.8 mm, BW = 460 Hz/Px, VENC (in-plane) = 0.6 m/s, VENC (through plane) = 1.0 m/s).

Blood pressure levels of the upper right arm were recorded before and after CMR examination after resting in a supine position for at least 5 min. Heart rate was documented every 4 min during 4D flow CMR.

### Data analysis

**Fig. 2:**
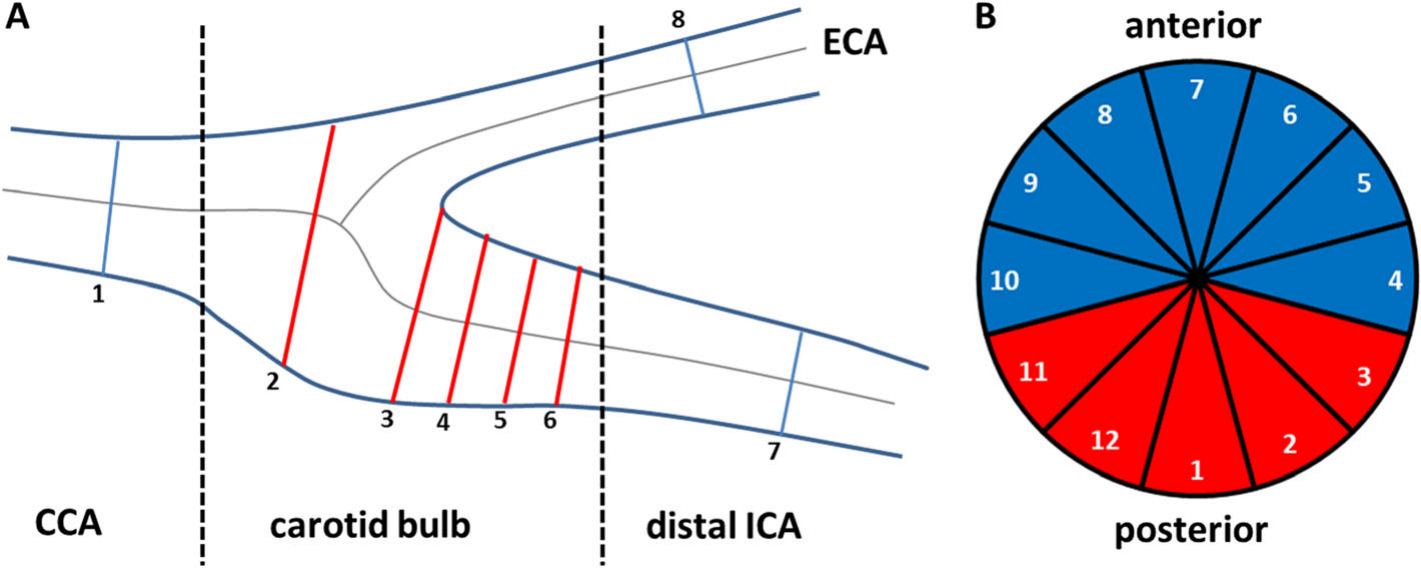
Schematic presentation of the standardized position of the 8 analysis planes along the carotid artery bifurcation (**a**). Each plane is divided into 12 wall segments (**b**), wall segments 1–3 and 11–12 of each of the planes 2–6 (labeled in red) represent the atherosclerosis prone region of the posterior carotid bulb. This scheme was used for both 4D flow and 3D-T1 weighted CMR data and the calculation of wall shear stress, oscillatory shear index and wall thickness values per plane and wall segment. CCA = common carotid artery; ICA = internal carotid artery; ECA = external carotid artery

For image processing data sets were imported into a custom-made extension (CaroTo) of the MEVISFlow research software (Fraunhofer MEVIS, Bremen, Germany [21]). Preprocessing of the 4D flow data included noise filtering, correction for Eddy currents and velocity aliasing. Then, a carotid artery mask (3D-phase-contrast CMR (PC-CMR)) was calculated from the 4D flow CMR data. In the next step, a centerline was created in each carotid bifurcation and landmarks (flow diverter and ICA) were marked. From these landmarks predefined points on the centerline were automatically generated, indicating the level of each analysis plane, which was initialized automatically in the next step. If necessary, analysis planes were manually oriented perpendicular to the carotid lumen. For quantitative image analysis we used a plane- and segment-based model as described in a previous study [8]. This was performed in 8 cross-section planes (Fig. 2a). The first plane was positioned on the CCA centerline 1 cm below the flow diverter. The second plane was placed within the ICA bulb, planes 3 to 6 along the ICA with the starting point at the flow diverter and oriented perpendicularly to the centerline with a spacing of each 3 mm. Plane 7 was the most distal ICA plane outside the plaque and positioned manually by the user. Plane 8 was placed automatically in the proximal external carotid artery (ECA). As described previously [8] each analysis plane was divided into 12 wall segments. Segment 1 was located at the posterior bulb (Fig. 2b). The remaining segments were numbered clockwise for the left and counterclockwise for the right carotid arteries. Plaques and stenoses are typically located in the distal CCA and proximal ICA at the posterior wall segments [7]. These were represented by segments 1–3; 11 and 12 in planes 2–6 (Fig. 2b), defined as atherosclerosis-prone region and used to evaluate the distribution of WSS parameters and of wall thickness. The required processing time of one 3D CMR dataset, including the analysis of bifurcation geometry, hemodynamics and wall thickness was 45–60 min.

### Geometry of the carotid bifurcation

**Fig. 3:**
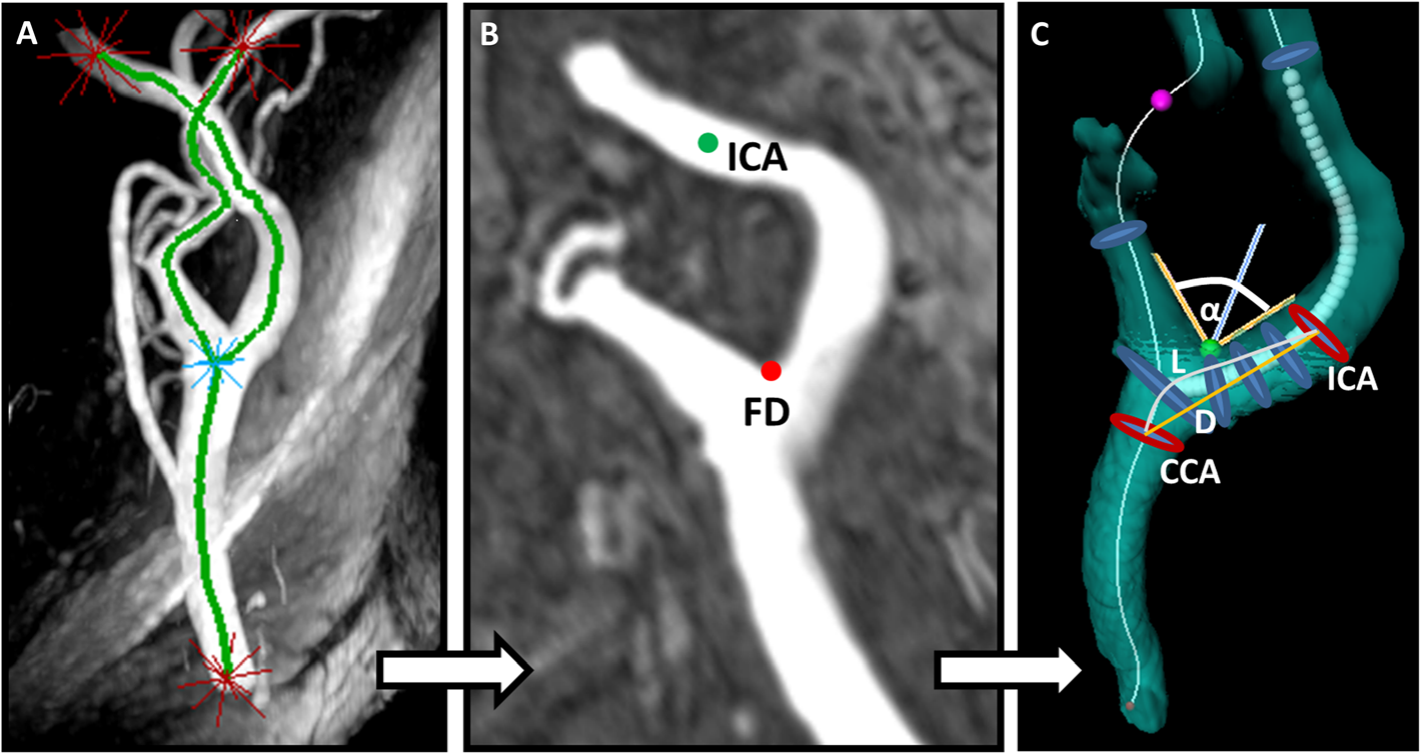
Post-processing workflow for the semi-automated assessment of carotid artery geometry. **a** Computation of a centerline based on time of flight (TOF)-CMR angiography after manual initialization of starting and end points (labelled as red stars). **b** Manual labeling of the ICA (green dot) and identification of flow diverter (FD, red dot). **c** Automatic computation of three geometry parameters: α indicates the bifurcation angle; ICA and CCA represent maximum diameters in plane 6 and plane1 for calculation of the ICA/CCA-ratio; L and D indicate the CCA-ICA distances along the lumen center (L) and the direct connection (D), respectively, for the calculation of carotid tortuosity. CCA = common carotid artery; ICA = internal carotid artery

Geometry of all 242 bifurcations was analyzed automatically based on the 3D-TOF CMR data after manually defining CCA, ICA, and ECA as start/endpoints for centerline computation and based on the location of the flow diverter (FD) as illustrated in Fig. 3. Geometric parameters were described and successfully used previously [8, 10]. They included a) ICA/CCA-diameter ratio, which was calculated using maximum ICA diameter in plane 6 and CCA diameter in plane 1, respectively; b) bifurcation angle, which was measured by two tangential lines of the first 1 cm of the outer wall starting at the FD; c) CCA-ICA tortuosity, which was assessed by calculating the ratio of the direct line and the centerline connection of the CCA in plane 1 and plane 6 in the ICA.

### Hemodynamic parameters

For each carotid bifurcation, the vessel-lumen boundary was manually outlined in the magnitude images of the 4D flow CMR data and propagated for each timeframe. Absolute WSS (in N/m^2^ = 1 Pa) was time-averaged over the cardiac cycle and derived for each vessel segment and for each plane. Systolic WSS was derived by averaging the time frames 2–5 in systole, which presumably contained peak systole. OSI (in %) was calculated as the degree of WSS inversion over the entire cardiac cycle as described previously [8].

### Wall thickness

**Fig. 4:**
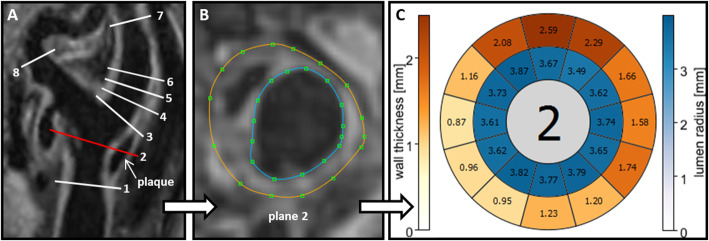
The post-processing concept for the semi-automated assessment of carotid wall thickness and lumen diameter is shown exemplarily in a patient with a calcified plaque in the carotid bulb. **a** Reconstructed longitudinal view of the carotid bifurcation based on a 3D-black-blood T1 weighted CMR-image with the standardized position of the eight analysis planes (see Fig. 2). **b** Manual segmentation of outer (brown line) and inner (blue line) vessel wall border in the cross-section corresponding to the red line. **c** Quantitative results of vessel lumen (inner ring labeled blue) and of wall thickness (outer ring labeled yellow to red) are displayed in a bulls-eye-plot comprising 12 evenly distributed wall segments

Post-processing of vessel wall thickness was based on 3D-T1-weighed CMR using the same custom-made software (CaroTo, Fraunhofer MEVIS, Bremen, Germany) comprising centerline computation after manual initialization and marking flow diverter and ICA. Quantitative assessment of vessel lumen and wall thickness was carried out in the same eight analysis planes and the 12 segments, which were positioned in the same fashion as described for WSS calculation. Wall thickness was measured after manual delineation of the inner and outer contours of the vessel wall (illustration of workflow in Fig. 4).

### Statistical analysis

Data are presented as mean and standard deviation or median (interquartile range) for continuous variables and as absolute frequencies and percentages for categorical variables. Departures from normality were detected with the Shapiro-Wilk and Kolmogorow-Smirnow statistics. Depending on data distribution two-tailed *t* tests or non-parametric tests were applied as appropriate for continuous variables.

In order to describe the distribution of wall thickness and hemodynamic parameters (absolute/systolic WSS and OSI) mean plots by side and stenosis are presented of the means in anterior and posterior segments. For each of these parameters a corresponding linear mixed model was performed with the respective parameter as dependent variable and a patient’s identity (ID) as random effect. Independent variables were anterior segment (yes/no), right side (yes/no), stenosis (yes/no, side specific), and planes 2–6 (see Fig. 2). In the following, the model corresponding to the primary outcome parameter “wall thickness” is called base model.

In order to investigate the impact of geometric (ICA/CCA-diameter ratio, bifurcation angle, tortuosity) and hemodynamic parameters (absolute/systolic WSS, OSI) on wall thickness, the base model was expanded with the respective parameters as independent variables. The base model was expanded to the final model by adding geometric and haemodynamic parameters while adjusting for risk factors (male gender, age, body mass index (BMI), diabetes mellitus, PAD, smoking, history of stroke or TIA, CAD, hypercholesteremia, hemoglobin A1c (Hb_A1c_) and low density lipoprotein (LDL)-cholesterol). Multiple measures in each patient were taken into account with the mixed effect in the model.

## Results

### Baseline characteristics

Table 1 summarizes the prevalence of cardiovascular disease risk factors and the number of patients with ≥10% ICA stenosis.

**Table 1:** Baseline characteristics of the study population

Characteristics	*N* = 121
**Age (years)**	**70.1 (±8.6)**
**Female sex - n (%)**	**38 (31.4)**
**Body mass index (kg/m**^**2**^**)**	**26.6 (±8.6)**
**Hypertension - n (%)**	**121 (100.0)**
**Diabetes mellitus - n (%)**	**39 (32.2)**
**Peripheral arterial disease - n (%)**	**14 (11.6)**
**Smoking habit - n (%)**	**32 (26.4)**
**Stroke/Transient ischemic attack - n (%)**	**21 (17.4)**
**Coronary heart disease - n (%)**	**31 (25.6)**
**Hypercholesterinemia - n (%)**	**84 (69.4)**
**Heart rate (beats/minute)**	**67.7 (±11.4)**
**Systolic blood pressure (mmHg)**	**145.8 (±16.6)**
**Diastolic blood pressure (mmHg)**	**80.7 (±10.9)**
**HbA**_**1c**_ **(mmol/l)**	**42.4 (±13.4)**
**LDL-cholesterol (mg/dl)**	**120.2 (± 51.8)**
**ICA stenosis ≥ 10% to ≤ 50% n (%)**	**45 (37.2)**

*TIA* transient ischemic attack, *HbA1c* hemoglobin A1c *ICA* internal carotid artery, *LDL* low density lipoprotein

### Carotid bifurcation geometry

All three geometric parameters were higher in the left compared to the right carotid bifurcation. In stenosis ≥10%, bifurcation angle and tortuosity were higher, the diameter ratio, however, was smaller compared to vessels without stenosis (Table 2).

**Table 2:** **A:** Geometric parameters in the left and right carotid arteries. **B:** Subgroup analysis of the same geometric parameters of all carotid arteries, but dichotomized into presence or absence of an internal carotid artery stenosis ≥10%

**A**	**Left carotid artery**	**Right carotid artery**	***P*** **value**
**Bifurcation angle [°]**	48.8 (37.8–69.6)	44.5 (31.1–64.3)	0.037
**Tortuosity**	1.06 (1.03–1.11)	1.04 (1.02–1.07)	0.005
**ICA/CCA-ratio**	0.76 (0.63–0.89)	0.73 (0.63–0.83)	0.018
**B**	**< 10% ICA stenosis**	**≥10% ICA stenosis**	***P*** **value**
**Bifurcation angle [°]**	46.3 (32.8–65.8)	45.4 (35.4–67.5)	0.015
**Tortuosity**	1.05 (1.03–1.09)	1.06 (1.03–1.10)	0.001
**ICA/CCA-ratio**	0.74 (0.67–0.87)	0.73 (0.59–0.87)	0.001

*CCA* common carotid artery, *ICA* internal carotid artery

### Distribution of wall shear stress

**Fig. 5:**
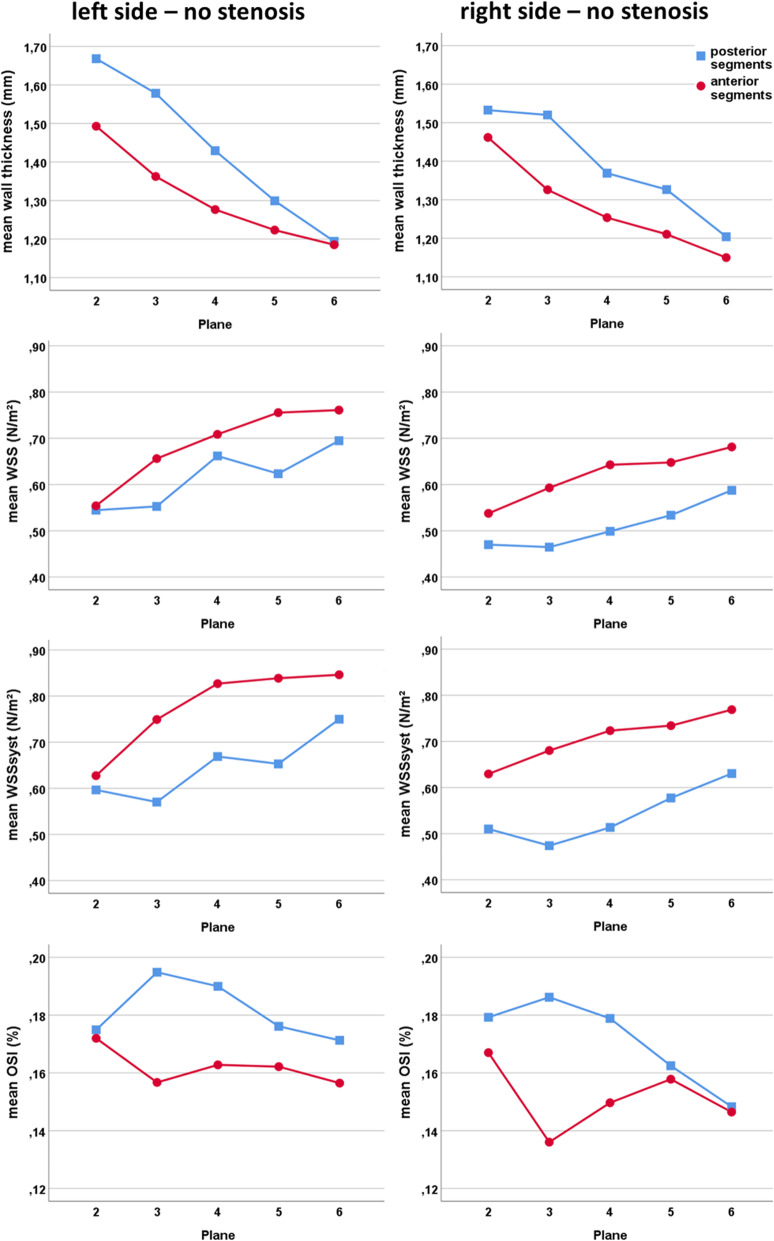
Distribution of wall thickness (upper row) and the three shear stress parameters (second to fourth row) of the left and right carotid bulb in patients without a stenosis

Mean absolute WSS and systolic WSS were significantly lower in the posterior than in the anterior wall of the bulb (planes 2–6, see Fig. 2) and lower in the right than in the left bifurcation (*p* < 0.001). Lowest values were found in the proximal bulb (=plane 2) with a slight increase downstream (Fig. 5). Values of absolute and systolic WSS were significantly higher in ≥10% ICA stenoses (*p* < 0.001), but still lower in the posterior wall compared to the anterior wall, especially in the right bifurcation (Additional file 1).

Inversely, OSI was higher in the posterior bulb (*p* < 0.001) and decreased significantly (*p* < 0.001) when stenoses were present. Highest values were found on the left side and in the proximal ICA bulb (=plane 2) (Fig. 5 and Additional file 1).

**Fig. 6:**
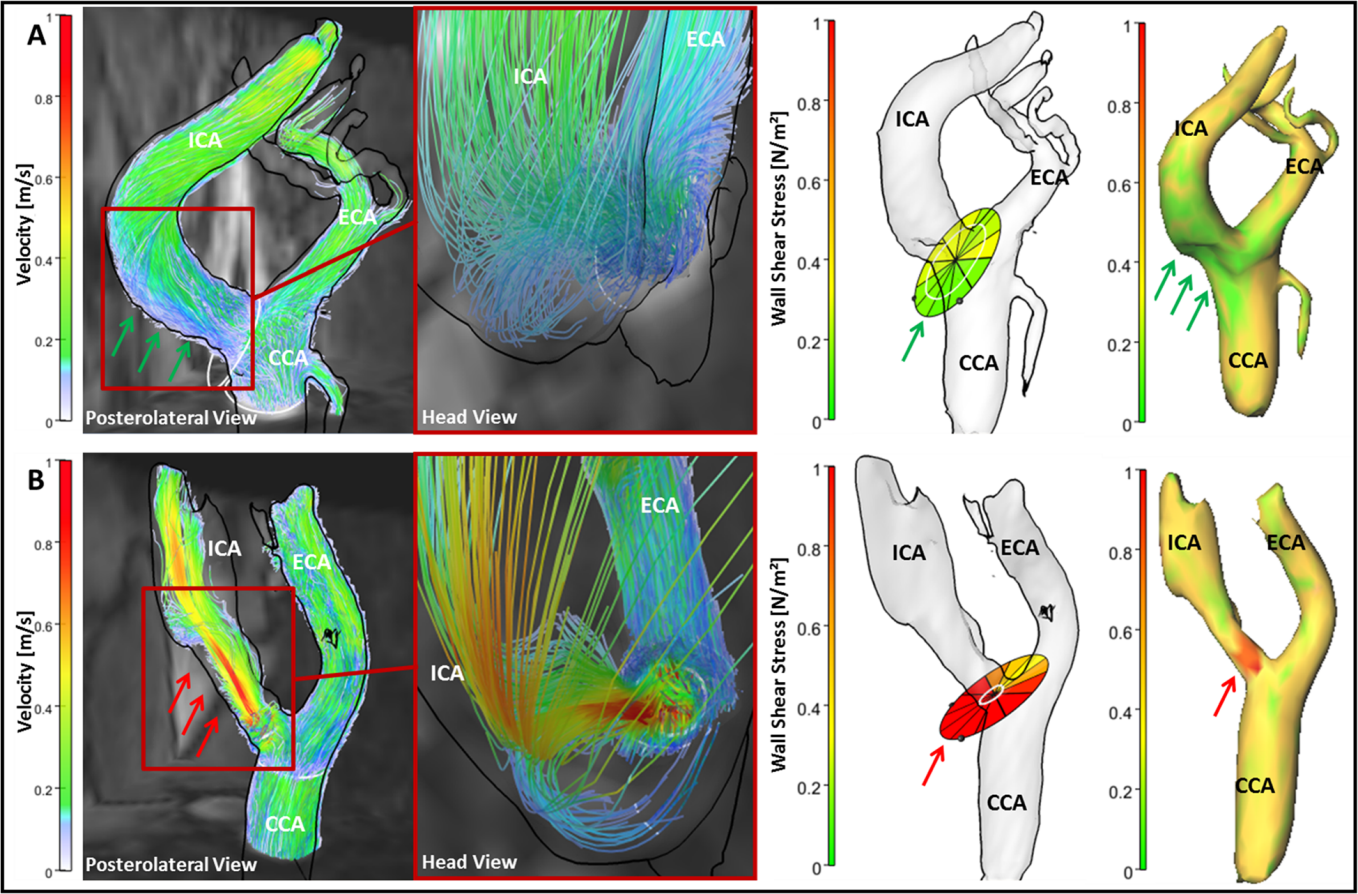
Blood flow velocity and wall shear stress parameters in a patient with a non-stenosing ICA plaque (**a**) and in a patient with a 40% ICA stenosis (**b**) in a posterolateral (first column) and head view (second column). Streamlines (left side) indicate lower velocity magnitudes in the absence of a stenosis (green arrows in **a**) resulting in lower wall shear stress (WSS) values in the posterior carotid bulb. These are exemplarily presented in plane 3 as segment-wise absolute WSS (third column) and as 3D maps of absolute WSS (fourth column). Presence of a stenosis resulted in a significant acceleration of blood flow velocities (first and second column, red arrows) and a consecutive rise in absolute WSS values (red arrow, third and fourth column). Color coding in first and second column represent absolute blood flow velocities in m/s. CCA = common carotid artery; ICA = internal carotid artery; ECA = external carotid artery. WSS = wall shear stress

Figure 6 exemplarily illustrates hemodynamic differences in a patient with an ICA plaque and in another with a 40% ICA stenosis, demonstrating distinct differences in 3D blood flow (i.e., blood flow acceleration and elimination of the helical flow pattern) and WSS distribution depending on the presence or absence of the bulb.

### Distribution of wall thickness

Wall thickness in the carotid bulb (planes 2–6) was significantly higher in posterior versus anterior wall segments, left versus right side, and in ≥10% stenoses versus ICA without stenoses (*p* < 0.001). Highest values were measured in the proximal bulb (=plane 2). Wall thickness decreased downstream of the ICA (Fig. 5 and Additional file 1).

### Impact of geometry, wall shear stress and cardiovascular risk factors on wall thickness

The ICA/CCA-diameter ratio was significantly and independently but inversely associated with vessel wall thickness (regression coefficient = − 0.302; *p* < 0.001). Accordingly, patients with thinner vessel wall had larger proximal ICA-diameters compared to those with thicker walls. In addition, vessel tortuosity was a significant and independent predictor of wall thickness in the carotid bulb (regression coefficient = 0.764, *p* < 0.001), even when adjusting for cardiovascular risk factors. However, we found no association of the bifurcation angle with wall thickness (regression coefficient = 0.000; *p* = 0.931). We neither identified an independent effect of absolute WSS, systolic WSS nor OSI on wall thickness when adjusting for risk factors.

Only male sex, but no other cardiovascular risk factor was independently related to increased wall thickness.

## Discussion

In this study, we investigated the impact of geometry and wall shear stress parameters on ICA wall thickness in 121 patients with advanced carotid atherosclerosis. Our CMR protocol allowed us to successfully acquire all parameters in 3D, with high spatial and temporal resolution and during one single examination lasting 45–60 min depending on patients’ heart rate. Key findings of our study were that lower absolute and systolic WSS and higher OSI were concentrated at the posterior bulb of the proximal ICA. So far, this has been only shown in studies of healthy subjects without preexisting atherosclerotic lesions and without a high cardiovascular risk factor load [8, 10]. In addition, bifurcation geometry and male sex were independent predictors of increased wall thickness of the ICA wall. However, WSS or OSI were not independent factors with regard to carotid wall thickness.

### Distribution of wall shear stress

Previous studies [7, 8, 10, 22] demonstrated the concentration of low absolute WSS or high OSI at the posterior wall of the ICA bulb in normal arteries, suggesting a causal connection of WSS and atherosclerosis, as this is the predilection site of carotid atheroma [7, 23, 24]. Consistently, we observed the same distribution in high-risk patients with carotid plaques. However, this pattern disappeared in ≥10% ICA stenosis due to the conversion of the ICA bulb into a straight or even stenotic segment by the plaque. Similarly, in case series of moderate to high-grade ICA stenosis, the typical WSS and OSI distribution disappeared and was restored after carotid surgery, respectively [8, 25]. Thus, our results support the hypothesis that low WSS and high OSI could influence the initiation of atherosclerosis, whereas in manifest stenosis other mechanisms, such as high maximum shear or mechanical stress may trigger plaque progression [16, 24, 26–28]. This concept is underlined by previous studies [13, 14] showing an association of high maximum shear stress with intra-plaque hemorrhage and conversion into a vulnerable plaque. Normal values or cut-off values for WSS or OSI predicting the individual risk of plaque development, progression or rupture are not yet available. In the past, arbitrary cut-offs representing 10% or 20% of the lowest or highest values in the respective study population were used. They differed according to study population, measurement accuracy (spatial/temporal resolution) and methods (CFD versus 4D flow CMR) [8, 10]. In the present study, we analyzed the distribution and local differences of absolute and mean values, which allow a direct comparison with future similar studies.

### Influence of geometry on carotid wall thickness

A large 2D-ultrasound study revealed that a dorsal/dorsomedial ICA outlet increased the likelihood of increased intima-media thickness of the ICA bulb [4], but was not able to assess geometry or wall thickness on a 3D level. This was later overcome by CTA [15], showing that decreased ICA-CCA diameter ratio and increased ICA angle were independently related to the degree of ICA stenosis. Finally, a 2D-CMR study [16] demonstrated that increased diameter of the bulb region and increased curvature, which is comparable to tortuosity [10], were independent but weak predictors of early wall thickening in the CCA and proximal ICA. Similar to Phan et al. [15] we observed an independent inverse relationship of the ICA/CCA diameter ratio and were able to correlate it directly to local wall thickness. This suggests that the ICA/CCA diameter ratio, thought to be one of the most influential predictors of disturbed flow [8, 10, 15, 16], loses its influence in patients with advanced atherosclerotic disease because of inward remodeling. Comparable to Bijari et al. [16], tortuosity was an independent predictor of local wall thickness in our study. The influence of tortuosity on the development of femoral artery atherosclerosis was also shown in 232 hyperlipidemic patients [29]. Surprisingly, increasing bifurcation angle, which is partly considered in the parameter tortuosity, representing changes of blood flow induced by geometry more adequately, was not an independent predictor for wall thickness, although having been correlated with WSS distribution and degree of ICA stenosis [8, 10, 15].

### Influence of shear stress on wall thickness

Healthy subject studies [8, 10] detected a concentration of atherogenic shear parameters in the ICA bulb, but only very limited data is available from studies [17, 30–32] investigating their influence on carotid wall thickness: in a study with 14 patients, phase-contrast CMR and CFD demonstrated that reduced WSS was inversely related to increased carotid wall thickness. However, influence of bifurcation geometry was not considered [17]. A 2D-ultrasound study of 48 patients showed that low WSS was an independent predictor of plaque progression over a 12-year period [33]. In addition, animal studies [11, 12, 34] impressively demonstrated that WSS parameters correlated with wall thickening, that low WSS led to the development of unstable plaques and high OSI formed stable plaques. However, animal models, as well as CFD studies, are not directly comparable to studies performed in humans, because of artificial induction of atherosclerotic lesions by a special diet in animal models and differences in vessel compliance and blood viscosity in CFD models.

In our study, we evaluated flow conditions and wall thickness under naturalistic conditions in 242 carotid bifurcations and directly compared WSS and OSI with wall thickness along the entire bifurcation. However, there was no independent association of WSS and OSI with carotid wall thickness. Since approximately 35% of patients had carotid stenoses ≥10%, we speculate that these hemodynamic parameters played an important role in early stages of atherosclerosis, whereas at the time of CMR measurement other factors may trigger progression of wall thickness and plaque size. Thus, following the literature and our results, the independent role of WSS or OSI for the formation of local atherosclerosis is still equivocal and requires further investigation in large-scale studies.

### Data acquisition and analysis strategy

Our protocol intra−/inter-observer agreement and reproducibility for WSS and OSI were good. In addition, measurement of carotid wall thickness employing a similar CMR protocol in patients [35, 36] was good to excellent for these items.

The comprehensive acquisition of 3D data of morphology and blood flow in one single CMR scan was realized with a reasonable spatial and temporal resolution. Post-processing software analysis automatically placed centerlines and analysis planes in a standardized manner in relation to anatomically defined markers. In addition, geometrical information was calculated automatically in contrast to previous in vivo studies [8]. This allowed for a minimization of measurement errors, an optimal comparison of geometry and WSS with wall thickness values and thus a comprehensive study of fluid-structure interaction in vivo.

### Limitations

We recruited patients with ≥2 cardiovascular disease risk factors and atherosclerotic plaques, including ≥10–50% ICA stenosis, allowing us to study different stages of atherosclerosis, but also resulting in a somewhat heterogeneous cohort, which may be one reason why WSS was not an independent predictor of local wall thickening. Alternatively, the measurements of WSS and OSI were performed at a too advanced stage of atherosclerosis. Also, a larger cohorts or follow-up measurements may be needed to detect the independent influence of WSS or OSI. Furthermore, based on our cross-sectional data, we are not able to state if individual geometry or WSS are predictors of future increase of local wall thickness, plaque growth, and rupture.

We used carotid wall thickness as dependent variable in our study. Plaque volume or plaque composition are other interesting parameters of carotid atherosclerosis that should be studied in their interaction with geometry and shear stress in the ICA bulb in a cross-sectional analysis and over time. This would be of high clinical interest as it is currently unclear if they provide additional or superior information compared to wall thickness. Unfortunately, a semi-automated quantification of plaque volume was not yet available but is currently under development and will be tested by our group in the near future.

Apart from the above-mentioned patient population-related effects, accuracy of blood flow and wall thickness measurements strongly depend on spatial and temporal resolution of the CMR data and require optimal image quality. Although our CMR protocol allowed consistent and standardized analysis, further improvements in resolution and reduction of wall motion through respiratory-, swallowing-, or ECG-gating may be beneficial and the duration of the examination may be reduced by new acceleration methods such as compressed sensing [37]. Processing of one dataset including the determination of geometry, hemodynamics and wall thickness required 45–60 min. Thus, our methods are well suited for research in smaller patient cohorts to study the underlying pathophysiology of atherosclerosis e.g. at the carotid bifurcation as done in the current study. However, due to the high effort of time our methods are not yet suited for the application in large-scale studies or clinical routine. Thus, further automatization and reduction of processing time to a few minutes are necessary.

## Conclusion

We found a distribution of atherogenic low WSS and high OSI values in the posterior region of the carotid bulb in patients with existing atherosclerosis. Presence of ICA stenosis altered this distribution due to the change of the shape of the ICA induced by plaque growth. High carotid tortuosity, low ICA-CCA diameter and male sex were independently associated with an increasing carotid wall thickness in the ICA bulb, indicating that male patients with elongated and wide ICA could be at particular risk of plaque existence, growth, and subsequent stroke in case of plaque rupture. In contrast, additional geometric parameters, WSS or OSI had no independent influence on wall thickness. Their influence and potential role is thus still unproven and needs to be investigated by further large and longitudinal studies in patients. 4D flow CMR and multi-contrast plaque CMR imaging have enormous potential in providing new risk stratification parameters, which could improve individualized treatment decisions with respect to the indication of e.g. vessel recanalization and ultimately patients’ outcomes. Previous CTA- and CMR studies [15, 16] have shown that carotid geometry independently correlates with carotid wall thickness. However, these studies were not able to also analyze 3D-blood flow including WSS and OSI. Thus, the novelty of our study is the ability to comprehensively study 3D fluid structure-interaction at the carotid bifurcation in vivo without the need of time consuming CFD. However, further improvements of processing speed and automatization of our analysis software are necessary before it can be use in large-scale population studies or in clinical routine.

## Supplementary information

**Additional file 1.** Distribution of wall thickness (upper row) and the three shear stress parameters (second to fourth row) in the left and right carotid bulb in patients with ≥10% and ≤ 50% ICA stenosis.

## Data Availability

The datasets used and/or analyzed during the current study are available from the corresponding author on reasonable request.

## Abbreviations

BMI: Body mass index
BW: Pixel bandwidth
CCA: Common carotid artery
CFD: Computational fluid dynamics
CAD: Coronary artery disease
CMR: Cardiovascular magnetic resonance
CTA: Computed tomography angiography
ECA: External carotid artery
ECG: Electrocardiogram
ETL: Echo train length
FD: Flow diverter
FOV: Field of view
HbA1c: Hemoglobin A1c
ICA: Internal carotid artery
LDL: Low density lipoprotein
NASCET: North American Symptomatic Endarterectomy Trial
OSI: Oscillatory shear index
PAD: Peripheral artery disease
PC: Phase contrast
PD: Proton density
SPACE: Sampling Perfection with Application optimized Contrasts using different flip angle Evolution
TA: Total acquisition time
TE: Echo time
TIA: Transient ischemic attack
TOF: Time of flight
TR: Repetition time
TSE: Turbo spin echo
WSS: Wall shear stress
