# Supplementary figures and images for: Carotid geometry is an independent predictor of wall thickness – a 3D cardiovascular magnetic resonance study in patients with high cardiovascular risk
Source: J Cardiovasc Magn Reson. 2020 Sep 10;22:67. doi: 10.1186/s12968-020-00657-5 (PMC7488078; doi:10.1186/s12968-020-00657-5)

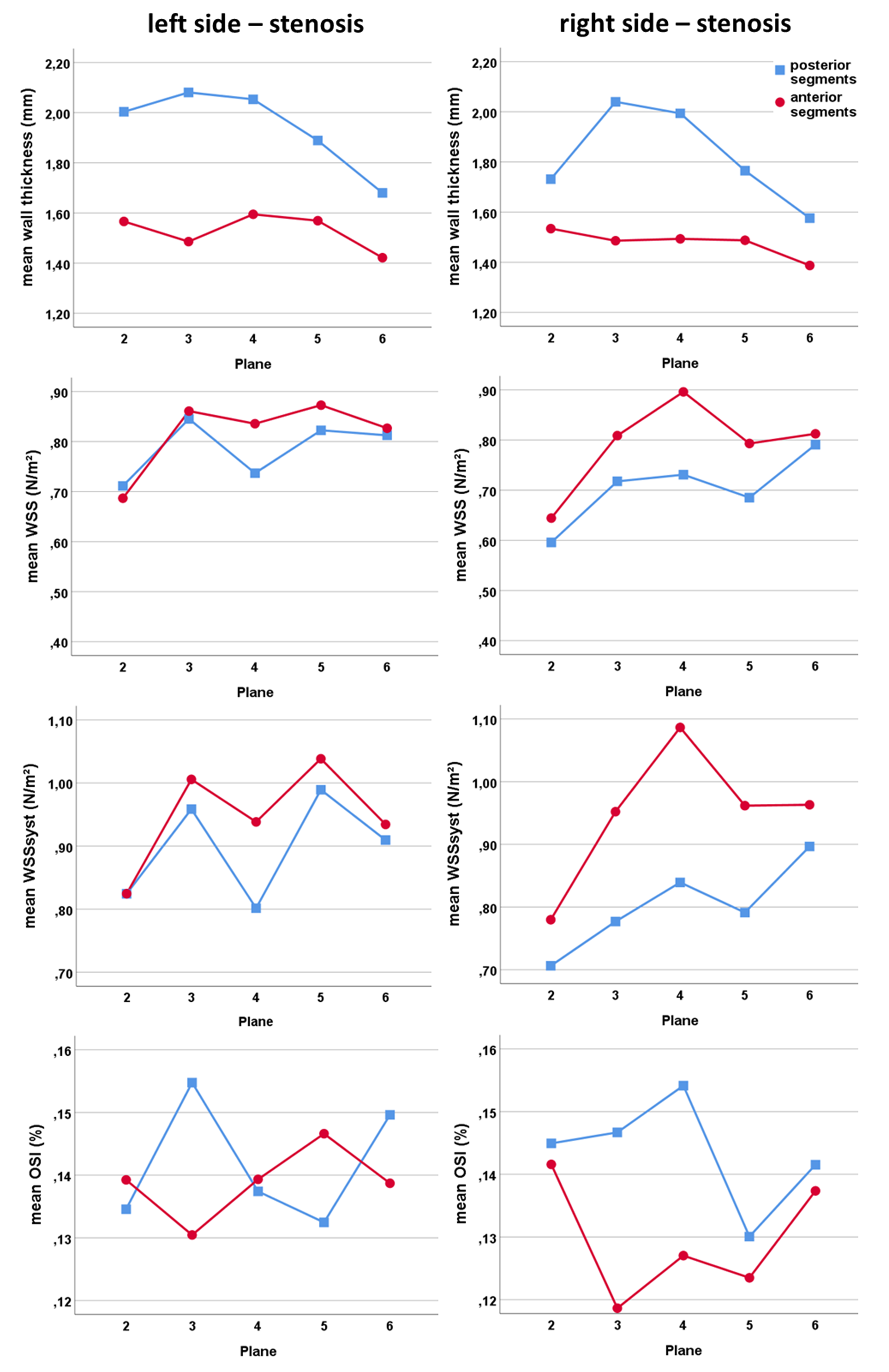

Supplement: Supplementary file 1 — Additional file 1. Distribution of wall thickness (upper row) and the three shear stress parameters (second to fourth row) in the left and right carotid bulb in patients with ≥10% and ≤ 50% ICA stenosis. [file 12968_2020_657_MOESM1_ESM.tif]
